# Die Rolle der farbkodierten Duplexsonographie zur präoperativen Gefäßdiagnostik in der plastischen Chirurgie

**DOI:** 10.1007/s00113-022-01166-z

**Published:** 2022-03-21

**Authors:** Adrian Matthias Vater, Rafael Jakubietz

**Affiliations:** Klinik und Poliklinik für Unfall‑, Hand‑, Plastische und Wiederherstellungschirurgie, Universitätsklinikum Würzburg, Oberdürrbacher Str. 6, 97080 Würzburg, Deutschland

## Originalpublikation

Vater AM, Prantl L, Noll M et al (2022) Gefäßdiagnostik vor mikrovaskulärem Gewebetransfer an der unteren Extremität. Unfallchirurg 125:66–72. 10.1007/s00113-021-00988-7.

## Erwiderung

Zum Leserbrief von Wahl U, Hirsch T (2022) Die zentrale Bedeutung der farbkodierten Duplexsonographie vor Planung eines mikrovaskulärem Gewebetransfers an der unteren Extremität. Unfallchirurg. 10.1007/10.1007/s00113-022-01164-1.

Dem Plädoyer der Kollegen Wahl und Hirsch für die farbkodierte Duplexsonographie (FKDS) als zuverlässiges Diagnostikum schließen sich die Autoren an. Die aufgeführten Daten untermauern den Stellenwert dieser Modalität in der Diagnostik von venösen und arteriellen Gefäßpathologien.

Der von den Autoren dargestellte Algorithmus hat zum Ziel, die präoperative Planung vor einem mikrochirurgischen Eingriff möglichst umfassend und effizient zu gestalten. Die Diagnostik der dem Defekt zugrunde liegenden Pathologie ist für den plastischen Chirurgen bedeutsam, jedoch für den Behandlungserfolg nicht allein entscheidend.

Neben der Detektion von Stenosen und Darstellung der Hämodynamik bedarf es zur Operationsplanung einer möglichst exakten topografischen Darstellung der Gefäßanatomie vom Körperstamm bis in die Peripherie. Insbesondere bei multimorbiden Patienten oder Patienten nach Trauma ist es aus plastisch-chirurgischer Sicht entscheidend, eine möglichst mehrdimensionale und realitätsnahe anatomische Darstellung zu erhalten, um mögliche Anschlussgefäße nicht nur identifizieren, sondern auch bei schwieriger Weichteilsituation muskuläre oder septale Verläufe sowie Plaques und Stenosen in ihrem Verlauf möglichst exakt und vollumfänglich darzustellen. Dies ist durch die CT-Angiografie (CTA) oder MR-Angiografie (MRA) und deren dreidimensionale Rekonstruktionsmöglichkeiten zuverlässig möglich und gewährleistet so eine sichere und effiziente Operationsplanung.

Vor allem bei der Darstellung schwerstkalzifizierter stenosierender Plaques, insbesondere bei langstreckigem Verlauf, zeigt die FKDS deutliche Schwächen im Vergleich zur CTA oder MRA [1–3]. Diese Informationen sind allerdings für die Evaluation einer präoperativen interventionell-radiologischen oder gefäßchirurgischen Intervention sowie für die mikrochirurgische Anastomosierung bei der Rekonstruktion unverzichtbar.

Hinzu kommt, dass durch die FKDS nicht immer eine ausreichend sichere Darstellung der infragenikulären Gefäße zu erzielen ist [1, 3, 4].

Entsprechend der zitierten Leitlinie muss die Auswahl von CTA und MRA trotzdem sorgfältig abgewogen werden. Die Anwendung ist demzufolge in Betracht zu ziehen, wenn die Planung eines gefäßchirurgischen Eingriffs infrage kommt [5]. Wenn auch nicht vom Gefäßchirurgen durchgeführt, so ist der mikrovaskuläre Gewebetransfer durch den plastischen Chirurgen hier zumindest synonym zu bewerten, von den Anforderungen an die umgebende Anatomie sogar deutlich komplexer.

Bei fehlendem klinischen Hinweis auf eine relevante Perfusionsstörung der unteren Extremität empfehlen die Autoren primär eine sonographische Gefäßdiagnostik, da hier im Regelfall nicht von einer wesentlichen Abweichung in der Anatomie und Topografie auszugehen ist und die FKDS – analog zu den Ausführungen von Wahl und Hirsch – eine ebenfalls sehr gute vaskuläre Diagnostik ermöglicht. Dadurch wird bereits eine Ressourcen-Schonung erzielt, da hier von vorneherein keine CTA oder MRA geplant wird. Sollte sich im sonographischen Befund jedoch ein Anhalt für anatomische Alterationen ergeben, empfehlen die Autoren dennoch eine CTA oder MRA, um vor dem geplanten Eingriff durch die Mehrinformation zu anatomischer Lage und Verlauf die Sicherheit des Eingriffs zu erhöhen.

Darüber hinaus bleibt festzustellen, dass der freie mikrovaskuläre Gewebetransfer selbst ein kostenintensiver operativer Eingriff ist. Daher gilt es, die Kosten und Ressourcen zur Operationsplanung dem operativen Erfolg entgegenzustellen. Hier genügt es aus Sicht der Autoren nicht, nur die Kosten für die Diagnostik zu betrachten. Misslingt aufgrund einer insuffizienten Planung der Eingriff, stehen die Kosten für Folgeeingriffe oder Folgen eines Extremitätenverlustes im Regelfall in keiner Relation zu der Kostendifferenz (104 € für die FKDS vs. 163 € für die CTA) in der präoperativen Diagnostik.

Die im Konsensuspapier erwähnten Faktoren wie Untersucherabhängigkeit und Zeitaufwand bei der FKDS stellen sich entgegen der Darstellung von Wahl und Hirsch im Klinikalltag für die Autoren dennoch als relevante Faktoren dar [6]. Für entsprechend ausgebildetes Fachpersonal in der vaskulären Ultraschalldiagnostik mögen Reliabilität und Zeitaufwand untereinander vergleichbar sein. Es ist jedoch naheliegend, dass nicht alle Untersucher in einem universitären Setting, insbesondere unter Ausbildungskriterien, DEGUM-Kriterien erfüllen können. Die Autoren erachten demzufolge die FKDS in der gesamten Breite gegenwärtig nicht uneingeschränkt als verlässlich [1, 3]. Selbiges gilt für die Untersuchungszeiten. Hier geben die Autoren zu bedenken, dass moderne hochauflösende Computertomographen eine Darstellung aller relevanter Strukturen binnen Sekunden generieren können, zudem benötigen erfahrene Radiologen nur wenige Minuten für eine entsprechende Befundung.

Zu betonen bleibt, dass der Stellenwert der FKDS in der präoperativen Gefäßdiagnostik keinesfalls geschmälert oder angezweifelt werden soll. Unter idealisierten Bedingungen könnten weitergehende Fragestellungen u. U. auch hierdurch beantwortet werden. Jedoch stehen die Vorteile der CTA oder MRA gegenüber der FKDS in für den plastischen Chirurgen wesentlichen Aspekten gegenwärtig außer Zweifel.
